# Prognostic and Monitoring Utility of Serum CEA in Lung Adenocarcinoma: Differential Roles in EGFR‐TKI and Chemotherapy Treatments

**DOI:** 10.1002/cam4.71170

**Published:** 2025-08-26

**Authors:** Yen‐Shou Kuo, Ming‐Yi Zheng, Yi‐Shing Shieh, Tsai‐Wang Huang, Yu‐Ting Chou

**Affiliations:** ^1^ Division of Thoracic Surgery, Department of Surgery Tri‐Service General Hospital, National Defense Medical University Taipei Taiwan; ^2^ Institute of Biotechnology National Tsing Hua University Hsinchu Taiwan; ^3^ Department of Dentistry Tri‐Service General Hospital Taipei Taiwan; ^4^ National Defense Medical University Taipei Taiwan

**Keywords:** biomarker, carcinoembryonic antigen (CEA), chemotherapy, epidermal growth factor receptor (EGFR), epithelial‐to‐mesenchymal transition (EMT), lung adenocarcinoma (LUAD), tyrosine kinase inhibitor (TKI)

## Abstract

**Background:**

Serum carcinoembryonic antigen (CEA) has potential prognostic and monitoring significance in lung adenocarcinoma (LUAD) patients undergoing different treatments, such as epidermal growth factor receptor (EGFR)‐tyrosine kinase inhibitors (TKIs) and chemotherapy. The changes in CEA expression during relapses, influenced by resistance mechanisms involving cytokines and epigenetic factors, may impact its utility in disease prognosis, monitoring, and management.

**Methods:**

This retrospective study analyzed advanced LUAD patients treated between 2011 and 2018, including 182 patients receiving EGFR‐TKIs and 102 undergoing chemotherapies. Serum CEA levels were measured at baseline and relapse. Associations between CEA levels, treatment modalities, and survival outcomes were assessed using Cox regression and Kaplan–Meier analyses. Gene expression profiling and in vitro experiments explored the regulation of CEACAM5 expression by cytokines and epigenetic mechanisms in EGFR‐TKI‐resistant cells.

**Results:**

Elevated baseline CEA (≥ 5 ng/mL) was associated with significantly worse overall survival (OS) in patients treated with EGFR‐TKIs but showed no prognostic value in chemotherapy‐treated patients. During the relapse, EGFR‐TKI–treated patients were more likely to exhibit a transition to CEA‐negative status (< 5 ng/mL) compared to those receiving chemotherapy. Mechanistic studies revealed that EGFR‐TKI‐resistant cells displayed reduced CEACAM5 expression and increased epithelial‐to‐mesenchymal transition (EMT) markers, driven by cytokine signaling and epigenetic modifications.

**Conclusions:**

Serum CEA is a stronger prognostic biomarker for LUAD patients treated with EGFR‐TKIs while offering consistent monitoring capabilities in chemotherapy‐treated patients. These findings highlight the differential clinical value of serum CEA in guiding therapeutic strategies and monitoring disease progression across treatment modalities.

## 1 Introduction

Lung adenocarcinoma (LUAD), a predominant subtype of non‐small cell lung cancer (NSCLC), is commonly treated with targeted therapies or chemotherapy, depending on the presence of specific driver mutations such as those in the epidermal growth factor receptor (EGFR) gene. EGFR‐tyrosine kinase inhibitors (TKI) have shown remarkable efficacy, particularly in tumors harboring *EGFR* exon 19 deletions (Del19) or exon 21 point mutations (L858R), which together represent approximately 90% of *EGFR* mutations [1]. Patients with these mutations typically experience prolonged progression‐free survival (PFS) when treated with EGFR‐TKI, compared to those receiving standard chemotherapy [2, 3, 4, 5, 6]. Studies report that PFS ranges from 10 to 19 months for patients treated with EGFR‐TKI, whereas those undergoing platinum‐based chemotherapy often have a shorter PFS of 4–6 months. Moreover, response rates to targeted therapies usually exceed 60%–70%, significantly higher than the 20%–30% observed with chemotherapy in the same patient population [4, 5, 6, 7, 8].

Carcinoembryonic antigen (CEA), initially identified as a tumor marker in colorectal cancer, is also expressed in various cancers, including LUAD [9]. Clinically, serum CEA levels are frequently used alongside imaging to monitor disease progression and predict survival outcomes, particularly in colorectal cancer [10, 11]. In early‐stage lung cancer, pre and postoperative CEA levels offer valuable insights into disease progression and the need for adjuvant therapy [12, 13]. However, the role of CEA in predicting survival and therapy sensitivity in advanced LUAD remains unclear.

Emerging evidence suggests an association between serum CEA expression and *EGFR* mutations in NSCLC, raising the possibility that CEA could have prognostic value in LUAD [14]. CEA has been explored as a surrogate marker for evaluating response to chemotherapy, and combining CEA with other serum biomarkers, such as lactate dehydrogenase (LDH) and CYFRA21‐1, has been shown to improve the prediction of chemotherapy response [15, 16]. However, CEA levels can be heterogeneous, varying before and after treatments such as EGFR‐TKI therapy or chemotherapy, with studies indicating drug‐induced plasticity in CEA expression [16, 17]. Despite this variability, CEA remains a widely used marker due to its low cost, noninvasive nature, and the lack of more reliable alternatives [18, 19].

The prognostic significance of CEA in advanced LUAD, particularly in comparing EGFR‐TKI therapy to chemotherapy, remains incompletely understood. Given the distinct mechanisms of action and variable response durations associated with these therapies, it is likely that CEA expression patterns may differ between treatment modalities. This study aims to investigate the prognostic value of CEA in advanced LUAD, comparing patients receiving first‐line EGFR‐TKI with those undergoing chemotherapy. Additionally, we explore the dynamic changes in CEA expression across these therapeutic settings, providing insights into how cancer cell heterogeneity evolves in response to different treatment strategies.

## 2 Materials and Methods

### 2.1 Patients and Samples

This study reviewed medical records of 4808 patients diagnosed with lung cancer, registered with the Cancer Registry Group at Tri‐Service General Hospital, National Defense Medical Center, between January 2011 and January 2018. Patients were excluded from the analysis if they had more than one primary cancer or lacked data on *EGFR* mutation status or CEA levels. Of the total, 2499 patients were identified with LUAD. Eligibility criteria included a histologically or cytologically confirmed diagnosis of stage IIIB–IV LUAD. Retrospective data were collected from 1271 patients who met these criteria, with a data cutoff for outcome analysis in June 2024. Among these, 676 patients had both baseline and relapse CEA data available for analysis. These 676 patients were subsequently grouped based on the presence or absence of *EGFR* mutations. Within the *EGFR* mutant group, 208 patients were identified, 182 receiving first‐line treatment with EGFR‐TKI. In contrast, 128 patients were categorized as having wild‐type EGFR, with 102 of them receiving chemotherapy as their first‐line treatment. The primary objective of this analysis was to explore the correlation between CEA levels and survival outcomes across these patient groups. This retrospective study was conducted with the approval of the Joint Institutional Review Board of Tri‐Service General Hospital (Approval Number: B202405162). The requirement for informed consent was waived due to the study's retrospective nature, and all methods were performed in compliance with relevant guidelines and regulations.

### 2.2 CEA Electrochemiluminescence Immunoassay and Chest Computed Tomography

Serum CEA levels were measured using an electrochemiluminescence immunoassay. This was performed on an automated analyzer (CEA‐RIACT, Cisbio Bioassays, France) during initial diagnosis and/or disease progression. By the National Health Insurance policy, chest computed tomography (CT) scans were performed every 3 months to monitor disease status.

### 2.3 *EGFR* Mutation Analysis

*EGFR* mutation analysis was conducted using tissue biopsy samples collected at diagnosis. The analysis was performed with the ROCHE cobas *EGFR* Mutation Test kit, identifying mutations in *EGFR* exons 19–21. Patients with detectable mutations in these exons were classified into the *EGFR*‐mutant group, while those without detectable mutations were assigned to the *EGFR* wild‐type group.

### 2.4 Patient Treatment

All eligible patients with stage IIIB‐IV LUAD and confirmed *EGFR* mutations received EGFR‐TKI, including gefitinib, erlotinib, or afatinib, as first‐line treatment during the study period. Upon developing resistance to EGFR‐TKI, most patients were treated with a platinum‐based chemotherapy regimen, commonly platinum plus pemetrexed. Patients who initially received chemotherapy as first‐line systemic therapy typically transitioned to other treatments, including immunotherapy or targeted therapies, depending on individual patient factors and tumor characteristics, after resistance to platinum‐based regimens developed.

### 2.5 Chemicals and Reagents

Recombinant human transforming growth factor‐beta (TGF‐β) was purchased from Sino Biological (Beijing, China). Romidepsin (FK228) was obtained from Med Chem Express (Monmouth Junction, NJ, USA).

### 2.6 Cell Culture

HCC827 (RRID:CVCL_2063) cells were kindly provided by Dr. Jeff Wang from the Development Center for Biotechnology (Taipei, Taiwan), while A549 cells were obtained from the American Type Culture Collection (ATCC) [20, 21]. Both HCC827 and A549 cells were cultured in RPMI‐1640 medium supplemented with 4 mM L‐glutamine, 1 mM sodium pyruvate, 10 mM HEPES, and 10% fetal bovine serum (FBS). The identity of both cell lines has been authenticated by short tandem repeat (STR) profiling within the past 3 years to ensure cell line integrity. HCC827 cells were treated with romidepsin (1 nM) for 3 weeks prior to quantitative real‐time PCR (qRT‐PCR) analysis of epithelial–mesenchymal transition (EMT) markers, including *CDH1* and *VIM*. Separately, both HCC827 and A549 cells were treated with TGF‐β (1 ng/mL) for 3 weeks, followed by qRT‐PCR assays to assess EMT marker expression.

### 2.7 qRT‐PCR

qRT‐PCR was conducted in triplicate using Universal Probe Library probes (Roche Applied Science) and gene‐specific primers on a StepOnePlus system (Applied Biosystems). RNA18S served as the housekeeping gene for normalization of the target gene expression levels. Gene expression was quantified using the 2^−ΔΔCt^ method to calculate relative changes in mRNA levels. Primer and probe sequences are listed in Supporting Information and Methods.

### 2.8 Public Domain Analysis

Public domain gene expression data were analyzed following previously described methods [22]. Public domain databases are listed in Supporting Information and Methods.

### 2.9 Statistical Analysis

Associations between serum CEA levels, *EGFR* mutation status, overall survival (OS), and progression‐free survival (PFS) were analyzed using Cox regression models. Characteristics between the EGFR therapy and chemotherapy groups were compared using Chi‐square tests for categorical variables (e.g., gender, stage, smoking status) and independent *t*‐tests for continuous variables (e.g., age, tumor size, body mass index [BMI]). Kaplan–Meier survival curves were generated to estimate OS and PFS for three groups: all patients (*n* = 284), patients receiving EGFR‐TKI therapy (*n* = 182), and patients receiving chemotherapy (*n* = 102). The log‐rank test was used to compare survival distributions between these groups. Box plots were created to display the distribution of initial CEA and relapse CEA levels within the two patient groups: those receiving targeted therapy (*n* = 182) and those receiving chemotherapy (*n* = 102). These box plots depicted the median, interquartile range (IQR), and potential outliers, providing a clear comparison between initial CEA and relapse CEA levels within each treatment cohort. Paired CEA levels were compared within each group using the Wilcoxon signed‐rank test. A two‐sided *p*‐value equal to or < 0.05 was considered statistically significant. Adjustments for multiple testing (multiplicity) were not applied to avoid reducing statistical power. All data analyses were performed using SPSS software, version 22 (IBM Corp., Armonk, NY).

## 3 Results

### 3.1 Patient Selection and Clinicopathological Features

**FIGURE 1:**
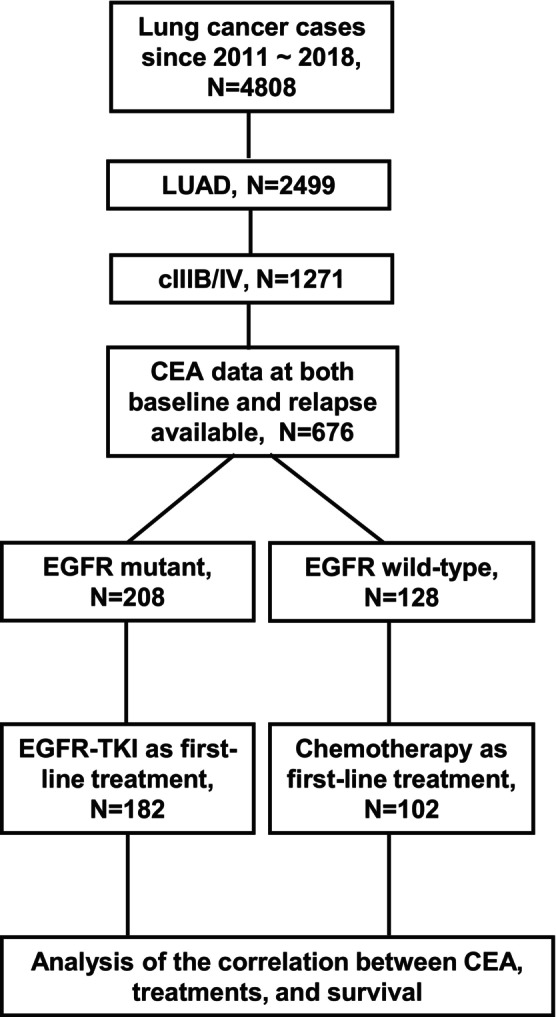
Patient selection flowchart for further analysis. This flowchart illustrates the patient selection process for the study. A total of 2499 lung cancer patients were initially identified from the cancer registry. Of these, 1271 were diagnosed with advanced‐stage LUAD. From this group, 676 patients with available baseline and relapse CEA data were selected. Among them, 208 patients had *EGFR*‐mutated LUAD, with 182 receiving first‐line treatment with EGFR‐TKI. In addition, 102 patients were diagnosed with *EGFR* wild‐type LUAD and received chemotherapy as their first‐line treatment. These patients were included in the subsequent correlation analysis of clinical characteristics with survival outcomes, including OS and RFS.

From a cohort of 4808 lung cancer patients registered at the Tri‐Service General Hospital, 2499 were diagnosed with LUAD. Of these, 1271 patients were classified as having advanced‐stage disease (clinical stages IIIB and IV). Serum CEA levels were available for 676 patients at both initial diagnosis and relapse. From this subset, 182 patients with confirmed *EGFR* mutations received EGFR‐TKI therapy as their first‐line treatment, while 102 patients without *EGFR* mutations underwent chemotherapy as their first‐line therapy. These 284 patients were selected for further analysis (Figure 1). The clinicopathological characteristics of the patients, including age, gender, clinical stage, tumor differentiation, tumor size, BMI, smoking history, Eastern Cooperative Oncology Group (ECOG) performance status, *EGFR* mutation status, baseline serum CEA levels, and relapse CEA levels, were detailed in Table 1. The median age of patients was 65 years (IQR: 53.6–76.4 years), with a median follow‐up duration of 21.1 months (IQR: 3.1–128.1 months). Among the 182 patients who received EGFR‐TKI therapy, 135 (74.2%) were nonsmokers, and 47 (25.8%) were ever‐smokers. In comparison, of the 102 patients treated with chemotherapy, 47 (46.1%) were ever‐smokers, and 55 (53.9%) were nonsmokers (Table 1). Overall, 64.1% (182/284) of the LUAD patients harbored *EGFR* mutations. Within the EGFR‐TKI cohort (*n* = 182), 83 patients (45.6%) had an *EGFR* exon 19 deletion, while 80 patients (44.0%) carried the L858R mutation. Overall, 64.1% (182/284) of the patients harbored EGFR mutations. Within the EGFR‐TKI group, the most common mutations were exon 19 deletion (83 patients, 45.6%) and L858R point mutation (80 patients, 44.0%). Regarding serum CEA levels, 72.5% (132/182) of patients in the EGFR‐TKI group had baseline CEA levels ≥ 5 ng/mL, compared to 64.7% (66/102) in the chemotherapy group. This difference reached statistical significance (*p* = 0.05), indicating that elevated baseline serum CEA levels are associated with *EGFR* mutation status. This dataset provides a well‐defined clinical comparison of LUAD patients receiving EGFR‐TKI therapy versus those treated with chemotherapy, enabling further evaluation of CEA as a prognostic and predictive biomarker.

**TABLE 1:** Clinical characteristics of patients with advanced lung adenocarcinoma eligible for further analysis.

Characters	All lung adenocarcinoma	Target therapy	Chemotherapy	*p*
	(*n* = 284)	(*n* = 182)	(*n* = 102)	
Age (mean ± SD)	65.01 ± 11.36	65.82 ± 11.41	63.57 ± 11.18	0.108
Age				
< 65	140 (49.3)	85 (46.7)	55 (53.9)	
≥ 65	144 (50.7)	97 (53.3)	47 (46.1)	
Gender				
Male	128 (45.1)	68 (37.4)	60 (58.8)	< 0.001
Female	156 (54.9)	114 (62.6)	42 (41.2)	
Stage				
III	25 (8.8)	7 (3.8)	18 (17.6)	
IV	259 (91.2)	175 (96.2)	84 (82.4)	
Tumor differentiation				
Well	16 (5.6)	12 (7.9)	4 (4.3)	0.024
Moderately	86 (30.3)	65 (42.8)	21 (22.6)	
Poorly	74 (26.1)	41 (27.0)	33 (35.5)	
Not mentioned	108 (38.0)	63 (34.6)	44 (34.6)	
Tumor size (mm, mean ± SD)	44.46 ± 20.92	46.77 ± 2.70	43.19 ± 1.61	0.227
BMI (mean ± SD)	23.28 ± 3.42	23.28 ± 3.25	23.29 ± 3.67	0.973
Smoking				
Never	190 (66.9)	135 (74.2)	55 (53.9)	0.001
Ever	94 (33.1)	47 (25.8)	47 (46.1)	
ECOG				
0	74 (37.8)	47 (25.8)	27 (26.5)	0.978
1	83 (42.3)	51 (28.0)	32 (31.4)	
2	27 (13.8)	16 (8.8)	11 (10.8)	
3	8 (2.8)	5 (2.7)	3 (2.9)	
4	4 (2.0)	3 (1.6)	1 (1.0)	
5	0 (0)	0 (0)	0 (0)	
*EGFR*				
Wild‐type	102 (35.9)	0 (0)	102 (100)	
Mutant	182 (64.1)	182 (100)	0 (0)	
18(18, 18&20)		7 (3.8)	—	
19(19, 19&20)		83 (45.6)	—	
20		7 (3.8)	—	
21(21, 19&21, 20&21)		80 (44.0)	—	
Other		5 (2.7)		
Baseline CEA				
< 5 ng/mL	86 (30.3)	50 (27.5)	36 (35.3)	0.05
≥ 5 ng/mL	198 (69.7)	132 (72.5)	66 (64.7)	
Relapse CEA				
< 5 ng/mL	95 (33.5)	65 (35.7)	30 (29.4)	0.258
≥ 5 ng/mL	189 (66.5)	117 (64.3)	72 (70.6)	

*Note:* Data are presented as median [25th and 75th percentiles].

Abbreviations: Baseline CEA, serum carcinoembryonic antigen level at initial diagnosis; BMI, body mass index; ECOG, Eastern Cooperative Oncology Group Performance Status Scale; EGFR, epidermal growth factor receptor; NA, not applicable; Relapse CEA, serum CEA level at disease recurrence.

### 3.2 Comparing the Prognostic Value of CEA in LUAD Patients With Distinct First‐Line Treatments

**FIGURE 2:**
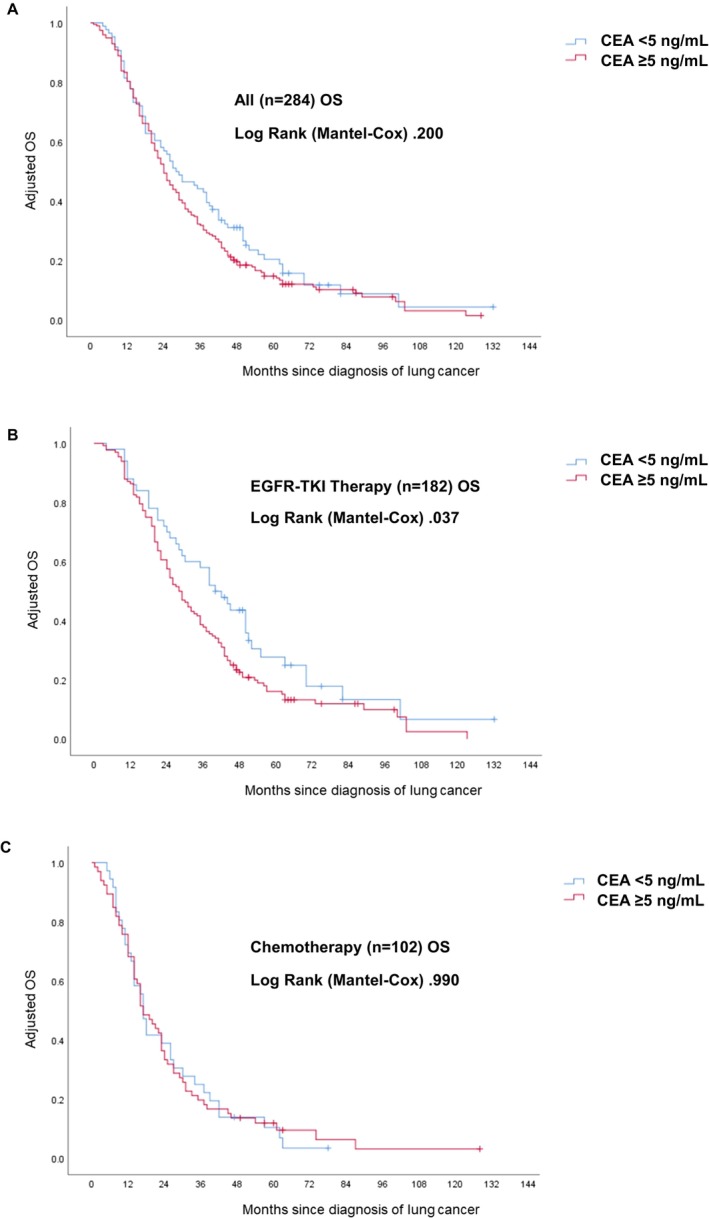
Effect of different first‐line treatments on CEA's prognostic role in patients with advanced‐stage LUAD. This figure illustrates adjusted OS based on a multivariable Cox model, analyzing the correlation between CEA expression and various first‐line treatments in patients with advanced‐stage LUAD. Panel (A) shows the analysis for all patients, while panel (B) focuses on those receiving EGFR‐TKI therapy, and panel (C) shows those receiving chemotherapy.

To investigate the prognostic significance of CEA levels in the survival of advanced‐stage LUAD patients, we performed a Kaplan–Meier analysis to evaluate the relationship between baseline serum CEA levels and OS in LUAD patients. The analysis showed that elevated CEA levels (baseline CEA ≥ 5 ng/mL) were not significantly associated with OS in the entire LUAD cohort (*n* = 284) (Figure 2A). However, in the subgroup of patients treated with EGFR‐TKI therapy (*n* = 182), high baseline CEA levels were associated with significantly poorer OS, a pattern not seen in the chemotherapy‐treated group (*n* = 102) (Figure 2B,C). Furthermore, elevated baseline CEA levels did not correlate with PFS in the overall cohort or the EGFR‐TKI or chemotherapy subgroups (Figure S1). These findings suggest that high serum CEA is an independent prognostic factor for poor OS, specifically in *EGFR*‐mutated LUAD patients treated with EGFR‐TKI therapy, but not in those treated with chemotherapy.

### 3.3 CEA as an Independent Prognostic Marker in LUAD Patients Receiving First‐Line EGFR‐TKI Treatment

To investigate the prognostic significance of CEA levels in the survival of advanced‐stage LUAD patients, we performed univariate and multivariable Cox regression analyses. These analyses compared CEA expression with other variables to independently predict survival outcomes in all patients with advanced LUAD (*n* = 284), as well as in subgroups receiving targeted therapy (*n* = 182) or chemotherapy (*n* = 102). In the overall cohort (*n* = 284), multivariable analysis identified stage and *EGFR* mutation status, but not smoking history or serum CEA levels, as independent factors associated with OS and recurrence‐free survival (RFS) (Table S1). *EGFR* mutations were linked to improved OS (adjusted hazard ratio [aHR] = 0.51, 95% confidence interval [CI]: 0.39–0.67, *p* < 0.001) but poorer PFS (aHR = 9.73, 95% CI: 5.52–17.14, *p* < 0.001). Serum CEA levels did not show prognostic value when considering both the targeted therapy and chemotherapy groups together. We further assessed the prognostic value of serum CEA levels, specifically in patients harboring *EGFR* mutations (*n* = 182) (Table 2). Multivariable analysis showed that elevated baseline CEA levels were an independent predictor of poor OS (aHR = 1.45, 95% CI: 1.00–2.10, *p* = 0.05). Additionally, the *EGFR* mutation subtype significantly predicted better RFS (Exon 19 Del: aHR = 0.164, 95% CI: 0.072–0.371, *p* < 0.001; Exon 20: aHR = 0.156, 95% CI: 0.051–0.472, *p* < 0.001; L858R: aHR = 0.169, 95% CI: 0.074–0.386, *p* < 0.001). To evaluate the prognostic role of serum CEA levels in advanced‐stage LUAD patients treated with chemotherapy as first‐line therapy (*n* = 102), we again conducted univariate and multivariable Cox regression analyses (Table S2). The multivariable analysis revealed that disease stage, but not smoking history or serum CEA levels, was an independent predictor of OS, with stage IV disease associated with worse OS (aHR = 2.317, 95% CI: 1.28–4.19, *p* = 0.006). Furthermore, age over 65 was linked to poorer RFS (aHR = 5.80, 95% CI: 1.59–21.08, *p* = 0.008). In contrast, baseline serum CEA levels did not have prognostic value in the *EGFR* wild‐type patient group treated with chemotherapy. These findings highlight the distinct prognostic value of serum CEA in LUAD patients undergoing EGFR‐TKI therapy, emphasizing its association with survival in those with *EGFR* mutations.

**TABLE 2:** Association between clinical characteristics and OS or PFS in 182 patients with advanced‐stage LUAD treated with first‐line EGFR‐TKI therapy.

Variable	Overall survival				Progression‐free survival			
	Univariate analysis		Multivariate analysis		Univariate analysis		Multivariate analysis	
	HR (95% CI)	*p*	aHR (95% CI)	*p*	HR (95% CI)	*p*	aHR (95% CI)	*p*
Age ≥ 65 years	1.060 (0.774, 1.453)	0.716	1.031 (0.747, 1.422)	0.854	1.090 (0.810, 1.466)	0.570	1.140 (0.829, 1.567)	0.420
Male sex	1.371 (0.991, 1.895)	0.056	1.338 (0.875, 2.045)	0.179	1.312 (0.965, 1.783)	0.083	1.156 (0.780, 1.714)	0.469
Stage IV	1.509 (0.618, 3.684)	0.367	1.630 (0.652, 4.076)	0.296	1.664 (0.736, 3.765)	0.222	1.778 (0.768, 4.115)	0.179
Ever smoking	1.343 (0.944, 1.911)	0.101	1.068 (0.674, 1.694)	0.778	1.351 (0.962, 1.897)	0.082	1.238 (0.807, 1.897)	0.328
*EGFR* mutant								
18(18,18&20)	Ref.		Ref.		Ref.		Ref.	
19(19,19&20)	0.545 (0.235, 1.260)	0.156	0.459 (0.195, 1.007)	0.073	0.193 (0.087, 0.429)	< 0.001	0.164 (0.072, 0.371)	< 0.001
20	0.526 (0.168, 1.646)	0.269	0.527 (0.164, 1.689)	0.281	0.149 (0.050, 0.451)	0.001	0.156 (0.051, 0.472)	0.001
21(21,19&21,20&21)	0.704 (0.305, 1.624)	0.410	0.620 (0.264, 1.456)	0.272	0.193 (0.087, 0.429)	< 0.001	0.169 (0.074, 0.386)	< 0.001
Other	0.265 (0.065, 1.072)	0.063	0.251 (0.062, 1.021)	0.054	0.058 (0.016, 0.205)	< 0.001	0.051 (0.014, 0.184)	< 0.001
Baseline CEA								
< 5 ng/mL	Ref.		Ref.		Ref.		Ref.	
≥ 5 ng/mL	1.461 (1.016, 2.101)	0.041	1.450 (1.000, 2.103)	0.050	1.172 (0.843, 1.631)	0.345	1.250 (0.883, 1.771)	0.208

Abbreviations: aHR, adjusted hazard ratio; CEA, carcinoembryonic antigen level in serum at disease diagnosis; CI, confidence interval; EGFR, epidermal growth factor receptor; HR, hazard ratio.

### 3.4 Impact of CEA Expression and Metastatic Pattern on Prognosis

Given the potential influence of metastatic patterns on survival outcomes, we evaluated the prognostic significance of baseline CEA levels and specific metastatic sites using a multivariable Cox proportional hazards model in stage IV LUAD patients (Table 3). Elevated baseline CEA levels (≥ 5 ng/mL) were independently associated with poorer OS (hazard ratio [HR] = 2.494; 95% CI: 1.266–4.914; *p* = 0.008). In contrast, first‐line treatment with EGFR‐TKIs was significantly associated with improved survival (HR = 0.393; 95% CI: 0.198–0.781; *p* = 0.008). The presence of brain metastasis also emerged as a strong negative prognostic factor (HR = 2.773; 95% CI: 1.511–5.088; *p* = 0.001). Other factors, including age > 65 years, male sex, bone metastasis, and liver metastasis, did not demonstrate statistically significant associations with OS (*p* > 0.05 for all). These findings underscore the prognostic value of both a serum biomarker (CEA) and metastatic pattern (particularly brain involvement), while also supporting the survival benefit of first‐line EGFR‐TKI therapy in patients with stage IV lung cancer.

**TABLE 3:** Multivariable Cox proportional hazards regression analysis of prognostic factors for overall survival in the stage IV lung cancer cohort (*n* = 259).

Variable	B	SE	Wald	df	*p*	Hazard ratio (exp[B])	95% CI for exp(B)
Age > 65	−0.439	0.303	2.094	1	0.148	0.645	0.356–1.168
Male	0.237	0.296	0.643	1	0.423	1.268	0.710–2.264
Initial CEA ≥ 5 ng/mL	0.914	0.346	6.974	1	0.008	2.494	1.266–4.914
First‐line EGFR‐TKI	−0.934	0.350	7.099	1	0.008	0.393	0.198–0.781
Brain metastasis	1.020	0.310	10.849	1	0.001	2.773	1.511–5.088
Bone metastasis	−0.253	0.332	0.582	1	0.446	0.777	0.406–1.487
Liver metastasis	0.998	0.517	3.725	1	0.054	2.712	0.985–7.471

### 3.5 Effect of CEA Expression on the Prognostic Impact of Distinct First‐Line EGFR‐TKIs

As early‐generation EGFR‐TKIs such as gefitinib may confer distinct prognostic outcomes, we further evaluated whether baseline serum CEA expression modifies the prognostic effect of first‐line EGFR‐TKI therapy—specifically comparing gefitinib with other EGFR‐TKIs—in patients with *EGFR*‐mutant lung cancer. Kaplan–Meier survival analysis showed that patients treated with first‐line gefitinib had significantly poorer OS compared to those who received other EGFR‐TKIs (Figure S2, upper; log‐rank *p* = 0.014). However, no significant difference in RFS was observed between the two groups (Figure S2, lower; log‐rank *p* = 0.992). To examine the potential interaction between baseline CEA levels and EGFR‐TKI type, we performed a Cox regression analysis incorporating an interaction term between high initial CEA (≥ 5 ng/mL) and first‐line gefitinib use. This interaction term showed a trend toward statistical significance (HR = 0.39; 95% CI: 0.15–1.05; *p* = 0.062) (Table S3), suggesting that elevated CEA levels may be associated with improved survival among patients treated with gefitinib. Kaplan–Meier analysis stratified by this interaction also demonstrated a similar trend (Figure S3; log‐rank *p* = 0.102), indicating a potential differential prognostic effect of high CEA levels depending on the EGFR‐TKI administered—particularly favoring gefitinib.

### 3.6 Differential CEA Heterogeneity Patterns in Patients Under EGFR‐TKI Therapy Versus Chemotherapy

**FIGURE 3:**
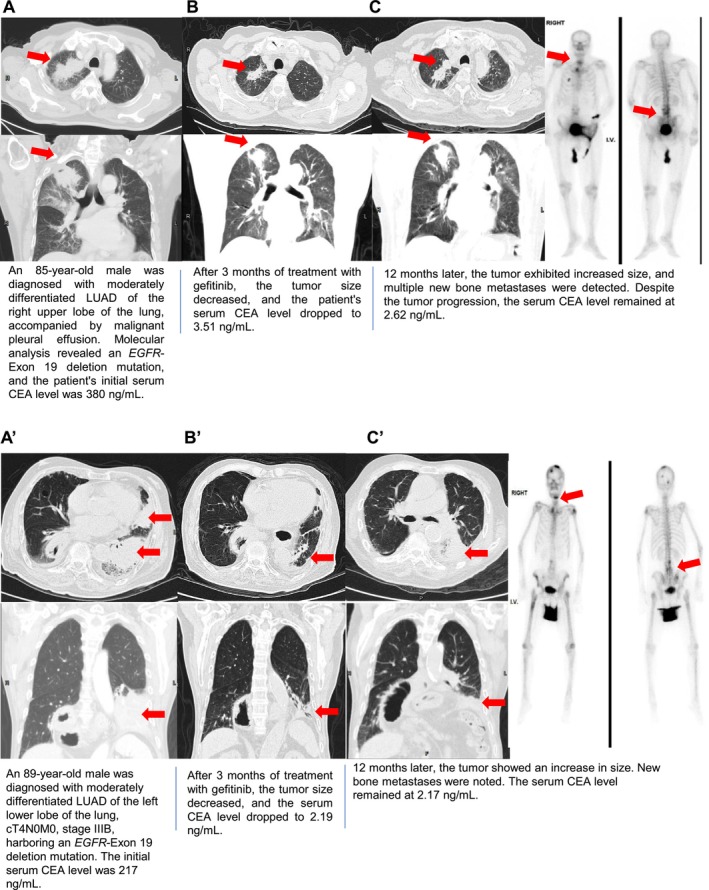
Serum CEA heterogeneity during EGFR‐TKI treatment in LUAD patients. This figure presents serum carcinoembryonic antigen (CEA) levels and imaging analyses at baseline, 3 months, and 12 months post‐gefitinib treatment in two patients (A–C and A'–C'). (A–C) An 85‐year‐old male with stage IV LUAD harboring an *EGFR* Exon 19 deletion mutation had an initial serum CEA level of 380 ng/mL. (A) Baseline imaging shows the primary tumor in the right upper lobe of the lung (red arrow). (B) After 3 months of gefitinib treatment, the tumor size reduced, and serum CEA levels decreased to 3.51 ng/mL. (C) After 12 months, the tumor progressed with an enlarged primary tumor and new metastases in the cervical and lumbar spine (red arrow), while serum CEA levels remained normal at 2.62 ng/mL. (A'–C') An 89‐year‐old male with stage IV LUAD harboring an *EGFR* Exon 19 deletion mutation had an initial serum CEA level of 217 ng/mL. (A') Baseline imaging shows the primary tumor in the left lower lobe of the lung (red arrow). (B') After 3 months of gefitinib treatment, the tumor size reduced, and serum CEA levels decreased to 2.19 ng/mL. (C') After 12 months, the tumor progressed with an enlarged primary tumor and new metastases in the cervical and lumbar spine (red arrow), while serum CEA levels remained normal at 2.17 ng/mL.

It has been suggested that CEA expression in LUAD patients may change between initial diagnosis and relapse, raising the possibility of using CEA as a marker for monitoring treatment response. To explore this, we investigated the dynamics of CEA expression in advanced‐stage LUAD patients undergoing either EGFR‐TKI therapy or chemotherapy by comparing baseline and relapse CEA levels before and after initial treatment. Our results demonstrated that CEA levels at relapse were significantly lower than baseline in the EGFR‐TKI therapy group, a pattern not observed in the chemotherapy group (Figure S4). Patients were categorized into two main groups based on their first‐line treatment (EGFR‐TKI or chemotherapy) and further subdivided into four cohorts according to their baseline and relapse CEA levels (< 5 ng/mL or ≥ 5 ng/mL) (Table 4). Notably, in Group 3, CEA‐positive patients (≥ 5 ng/mL) were more likely to convert to CEA‐negative (< 5 ng/mL) following EGFR‐TKI therapy compared to chemotherapy‐treated patients (Fisher's exact test, *p* = 0.039). Additionally, two LUAD patients with Exon‐19 deletions and high baseline CEA levels (> 100 ng/mL) were treated with gefitinib as first‐line therapy (Figure 3). CEA levels, CT scans, and bone scans were monitored at 3 and 12 months of EGFR‐TKI treatment. After 3 months, tumor shrinkage was observed, with CEA levels dropping below 5 ng/mL. However, by 12 months, tumor relapses and new bone metastases were detected, even though serum CEA levels did not rise. These findings suggest that EGFR‐TKI therapy is more likely than chemotherapy to induce a transition from CEA‐positive to CEA‐negative status in LUAD patients, underscoring both the challenges and the potential of using CEA as a marker for monitoring treatment response in patients receiving EGFR‐TKI therapy versus chemotherapy.

**TABLE 4:** CEA heterogeneity in LUAD patients treated with EGFR‐TKI therapy versus chemotherapy.

Baseline CEA versus relapse CEA (ng/mL)	< 5, < 5	< 5, ≥ 5	≥ 5, < 5	≥ 5, ≥ 5
	Group 1	Group 2	Group 3	Group 4
Targeted therapy (*n* = 182)	37 (20.3)	13 (7.1)	30 (16.5)	102 (56.0)
Chemotherapy (*n* = 102)	20 (19.6)	16 (15.7)	8 (7.8)	58 (56.9)
CEA heterogeneity	No	Yes	Yes	No

*Note:* P of Fisher's exact test = 0.039.

### 3.7 Regulation of CEA Expression Plasticity by Cytokine and Epigenetics Stimulation

**FIGURE 4:**
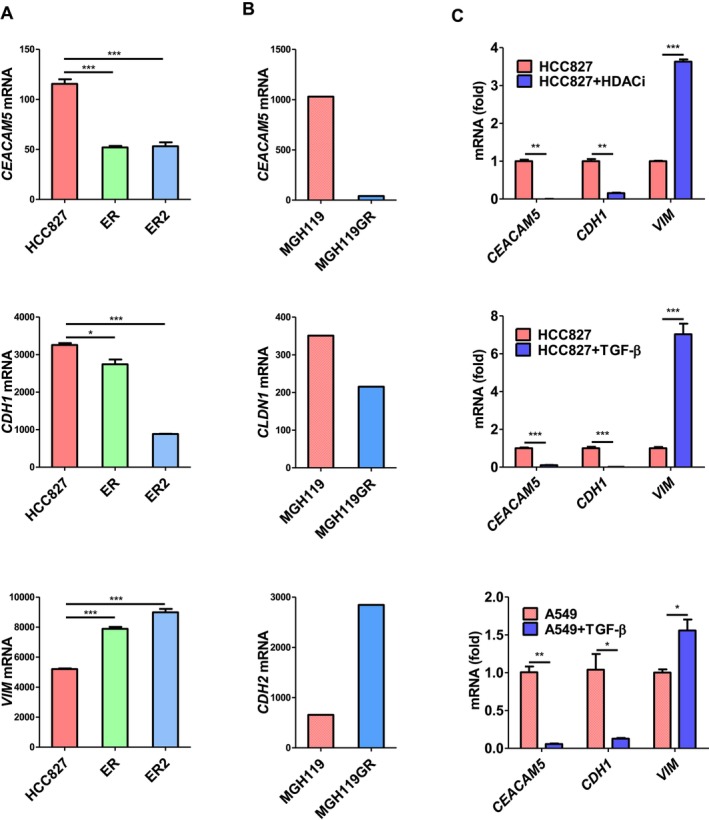
CEACAM5 expression plasticity is regulated by EGFR‐TKI selection and mediated by epigenetic and cytokine stimulation. (A) Gene expression profiling analysis evaluating *CEACAM5, CDH1, and VIM* expression in EGFR‐TKI sensitive HCC827 versus resistant counterpart HCC827ER1 (ER1) and HCC827ER2 (ER2) cells from the GSE38310 database. *p < 0.05; ***p < 0.001. (B) Gene expression profiling analysis evaluating *CEACAM5*, the epithelial marker gene *CLDN1*, and the mesenchymal marker gene *CDH2* and *VIM* expression in EGFR‐TKI sensitive primary tumor cells (MGH119) versus resistant (MGH119GR) cells from the GSE64322 database. (C) qRT‐PCR analysis assessing *CEACAM5*, *CDH1*, and *VIM* expression in HCC827 cells treated with or without romidepsin (HDACi, 1 nM) for 3 weeks. qRT‐PCR analysis evaluating *CEACAM5*, *CDH1*, and *VIM* expression in HCC827 cells treated with or without TGF‐β (1 ng/mL) for 3 weeks. qRT‐PCR analysis evaluating *CEACAM5*, *CDH1*, and *VIM* expression in A549 treated with or without TGF‐β (1 ng/mL) for 3 weeks. *p < 0.05; **p < 0.01; ***p < 0.001.

EGFR‐TKI resistance in LUAD cells is partly attributed to EMT, regulated by cytokine and epigenetic stimulation. To investigate whether EGFR‐TKI treatment affects CEA expression plasticity accompanied by EMT, we conducted gene expression profiling analyses to compare *CEACAM5* (the gene encoding CEA) and EMT marker gene expression in the LUAD cell line HCC827 and its EGFR‐TKI‐resistant clones (Figure 4A). Our findings revealed that the EGFR‐resistant clones, ER and ER2, selected by erlotinib, exhibited lower *CEACAM5* and epithelial marker *CDH1* expression but higher mesenchymal marker *VIM* expression compared to their parental HCC827 cells. Similarly, primary *EGFR*‐mutated lung cancer cells (MGH119) showed higher CEACAM5 and epithelial marker CLDN1 expression but lower mesenchymal marker CDH2 expression than their EGFR‐TKI gefitinib‐resistant counterparts (MGH119GR) (Figure 4B). These results suggest that EGFR‐TKI selection enriches LUAD cells with low CEACAM5 expression and high EMT features. To explore the role of epigenetic regulation in *CEACAM5* expression plasticity, HCC827 cells were treated with the HDAC1/2 inhibitor romidepsin for 3 weeks. Subsequent qRT‐PCR analysis showed that romidepsin treatment reduced the expression of *CEACAM5* and *CDH1* while increasing *VIM* expression, indicating that epigenetic mechanisms contribute to *CEACAM5* plasticity (Figure 4C, top). Additionally, as TGF‐β influences epigenetics and induces EMT and EGFR‐TKI tolerance, we treated *EGFR*‐mutated HCC827 cells and wild‐type A549 LUAD cells with TGF‐β. qRT‐PCR analysis revealed that TGF‐β stimulation downregulated *CEACAM5* and induced EMT in both cell lines (Figure 4C, middle and bottom). These findings suggest that CEA plasticity during the development of EGFR‐TKI resistance could be influenced by cytokine and epigenetic regulation, highlighting the complex interplay between these factors in modulating CEA expression plasticity and EGFR‐TKI resistance in LUAD cells.

## 4 Discussion

CEA is widely used to monitor disease progression and treatment response in various cancers, including LUAD [13, 19, 23]. However, the prognostic significance of serum CEA levels in LUAD patients undergoing either EGFR‐TKI therapy or chemotherapy remains a crucial area of clinical investigation, mainly due to the scarcity of reliable and easily applicable biomarkers. Notably, few studies have systematically evaluated the prognostic and monitoring value of serum CEA levels—both at baseline and in response to treatment—within the same patient cohort across different therapeutic modalities [24]. Our study highlights the critical role of personalized management strategies, emphasizing the value of baseline CEA levels and dynamic changes in CEA trends as important factors for guiding treatment decisions and improving patient outcomes.

Several studies have suggested that baseline CEA levels can serve as a prognostic marker associated with poor survival outcomes in NSCLC patients following chemotherapy or surgery. However, conflicting evidence exists, as some studies have reported that baseline CEA does not predict survival outcomes [25, 26, 27, 28, 29, 30, 31]. One study found that a high baseline CEA level (≥ 5 ng/mL) was linked to poor post‐operative survival in stage I‐IIIA NSCLC patients, although the study did not evaluate the prognostic value of CEA based on *EGFR*‐wild and *EGFR*‐mutant subgroups [32]. Another study demonstrated that a high baseline CEA level (> 10 ng/mL) was associated with poor OS and PFS in stage IIIB/IV NSCLC patients with *EGFR* mutations, yet this association was not explored in the *EGFR*‐wild cohort [33]. Our findings show that elevated baseline serum CEA levels (≥ 5 ng/mL) are significantly associated with poorer OS in stage III‐IV patients with *EGFR*‐mutated LUAD undergoing EGFR‐TKI therapy. This indicates that high serum CEA levels may serve as a negative prognostic indicator in this patient group, potentially informing the intensity and frequency of follow‐up care. Notably, the prognostic value of serum CEA was independent of clinical factors such as age, sex, and smoking status, underscoring its robustness as a prognostic marker in the context of first‐line EGFR‐TKI treatment. Conversely, our study found that baseline serum CEA levels did not exhibit significant prognostic value for OS or PFS in patients receiving chemotherapy as a first‐line treatment. This aligns with previous reports, which highlight the limitations of serum CEA in accurately predicting chemotherapeutic efficacy when used as a standalone biomarker [15, 16].

Previous studies have shown that serum CEA levels are more elevated in *EGFR*‐mutated NSCLC patients compared to those with wild‐type EGFR and can predict the efficacy of EGFR‐TKI treatment in NSCLC harboring *EGFR* mutations [14, 23, 34, 35]. Chiu et al. found that higher baseline CEA levels were associated with improved treatment response and survival in advanced NSCLC patients receiving gefitinib [36]. More recently, Uysal et al. demonstrated a significant association between elevated pretreatment CEA levels and *EGFR* mutation status in advanced LUAD [37]. Similarly, Liu et al., analyzing a large Chinese cohort, confirmed that higher serum CEA levels were significantly correlated with *EGFR*‐mutant genotypes, supporting the use of CEA as a potential noninvasive biomarker for genotype prediction [38]. In our own dataset, we observed a higher proportion of patients with baseline CEA levels ≥ 5 ng/mL in the *EGFR*‐mutant group compared to the wild‐type group, in line with the findings from these previous studies. Notably, these associations appear more pronounced in Asian populations. Liu et al. attributed this observation to the higher prevalence of *EGFR* mutations among Asians compared to Caucasians, suggesting that ethnicity‐related differences in tumor biology and serum marker expression may underlie this phenomenon.

Beyond lung cancer, members of the CEA family also serve as important biomarkers in breast cancer [39]. Immunohistochemistry analyses have demonstrated that CEACAM5 expression varies among breast cancer subtypes. CEACAM5 is expressed in more than 60% of non‐triple‐negative breast cancers (luminal and HER2‐enriched subtypes), while fewer than 30% of triple‐negative breast tumors show CEACAM5 expression [39]. Furthermore, high CEACAM6 expression is predominantly associated with luminal and HER2‐enriched breast cancer subtypes compared to basal‐like subtypes [40]. Gene expression profiling has also revealed that high CEACAM5 expression predicts poor outcomes in estrogen receptor (ER)‐positive breast cancer, while low CEACAM5 expression is linked to poor survival in basal‐like breast cancer [39]. Similarly, immunohistochemistry studies have shown that CEACAM5 is highly expressed in more than 50% of NSCLC tumors [41]. Moreover, elevated baseline CEA levels were associated with poorer OS in *EGFR*‐mutant LUAD patients receiving EGFR‐TKI therapy, further supporting the prognostic relevance of serum CEA in this molecular subtype. In contrast, no such association was observed in EGFR‐wild‐type patients treated with chemotherapy. Together, these findings suggest that CEACAM5 expression and its serum surrogate marker, CEA, may serve as subtype‐specific prognostic or predictive biomarkers in both lung and breast cancers, particularly in tumors driven by EGFR mutations or enriched in luminal and HER2‐related signaling pathways.

Previous studies have reported that early‐generation EGFR‐TKIs, such as gefitinib, may exhibit inferior efficacy as first‐line targeted therapy in patients with *EGFR*‐mutant [42]. Consistent with these findings, we observed that patients receiving first‐line gefitinib had significantly poorer OS compared to those treated with other EGFR‐TKIs, although no significant difference in RFS was noted between the two groups. Interestingly, Cox regression analysis incorporating an interaction term between elevated baseline CEA levels (≥ 5 ng/mL) and first‐line gefitinib treatment showed a trend toward improved survival in this subgroup, though it did not reach statistical significance (HR = 0.39; 95% CI: 0.15–1.05; *p* = 0.062). Supporting this observation, Kaplan–Meier analysis stratified by both CEA status and EGFR‐TKI type demonstrated a similar trend, indicating a potential differential prognostic impact of elevated CEA levels depending on the specific EGFR‐TKI used—particularly favoring gefitinib. While gefitinib was associated with poorer OS in the overall cohort, our interaction analysis suggests that it may attenuate the negative prognostic influence of elevated baseline CEA levels. These findings raise the possibility that serum CEA could function as a predictive biomarker for gefitinib responsiveness. Mechanistically, our recent study demonstrated that CEACAM6 mediates mutant EGFR signaling by directly interacting with and stabilizing the receptor [43]. It is plausible that high serum CEA levels may reflect tumor dependence—or “oncogene addiction”—on mutant EGFR signaling. In this context, tumors with elevated CEA expression may remain sensitive to gefitinib, despite its lower dosing and potency compared to later‐generation EGFR‐TKIs. This mechanistic insight may explain the observed trend toward improved survival among patients with high CEA levels who received gefitinib.

In addition to serving as biomarkers, CEA family proteins play critical roles in driver‐mediated oncogenesis. For instance, CEACAM6 interacts with HER2 in breast cancer cells, and CEACAM6 knockdown reduces the inhibitory effects of the anti‐HER2 antibody trastuzumab in trastuzumab‐sensitive cells [44]. Similarly, CEACAM6 interacts with EGFR to modulate signaling in oral cancer upon ligand activation [45]. These findings highlight the potential involvement of CEA family proteins in EGFR‐targeted therapies, which may explain their ability to predict prognosis in patients receiving EGFR‐targeted treatments. In contrast, the absence of an association between CEA levels and survival outcomes in chemotherapy‐treated patients could be due to the differing mechanisms of action between chemotherapy and EGFR‐targeted therapies. While EGFR‐targeted treatments directly engage specific molecular pathways associated with CEA family proteins, chemotherapy operates through broader cytotoxic mechanisms, which might explain CEA's lack of predictive value in this context.

It has been reported that distinct metastatic patterns influence prognosis in patients with *EGFR*‐mutant LUAD. For instance, Wu et al. demonstrated that liver metastases were associated with significantly poorer survival in patients receiving first‐line gefitinib therapy [46]. In contrast, Beypinar et al. observed no significant difference in the distribution of metastatic sites between *EGFR*‐mutant and wild‐type patients, yet paradoxically reported improved OS in those with liver metastasis [47]. Similarly, Hendriks et al. found comparable incidences of brain and bone metastases between *EGFR*‐mutant and wild‐type patients, but noted that post‐metastatic survival following bone involvement was significantly longer in the *EGFR*‐mutant cohort [48]. In our study, we assessed the prognostic impact of baseline CEA levels and metastatic site involvement in patients with stage IV LUAD using a multivariable Cox proportional hazards model. Elevated baseline CEA levels (≥ 5 ng/mL) were independently associated with inferior OS, highlighting its potential as a clinically relevant serum biomarker. Conversely, first‐line treatment with EGFR‐TKIs conferred a significant survival benefit, consistent with their established efficacy in *EGFR*‐mutant LUAD. Among the metastatic sites evaluated, brain metastasis emerged as a strong negative prognostic factor (HR = 2.773; 95% CI: 1.511–5.088; *p* = 0.001). While bone and liver metastases did not reach statistical significance, trends were observed: bone metastasis was associated with a nonsignificant favorable trend (HR = 0.777; 95% CI: 0.406–1.467; *p* = 0.446), whereas liver metastasis showed a borderline association with worse survival (HR = 2.712; 95% CI: 0.985–7.471; *p* = 0.054). Collectively, these findings underscore the prognostic relevance of both baseline serum CEA levels and specific metastatic patterns—particularly brain involvement—in stage IV LUAD. Furthermore, they reaffirm the survival advantage conferred by first‐line EGFR‐TKI therapy in this patient population.

CEA heterogeneity trends play a significant role in treatment decision‐making and patient outcomes. Our findings showed that CEA‐positive patients receiving chemotherapy were less likely to revert to CEA‐negative status upon relapse, while CEA‐negative patients were more likely to convert to CEA‐positive after relapse, compared to those under EGFR‐TKI therapy. These dynamics suggest that CEA may be a more reliable biomarker for monitoring patients treated with chemotherapy than those receiving EGFR‐TKI therapy. Furthermore, CEA expression plasticity has been shown to influence the efficacy of cibisatamab, a T cell bispecific antibody that links CEA on cancer cells to CD3 on T cells [49]. Treatment with cibisatamab was found to enrich cancer cells with deficient *CEACAM5* expression due to the activation of the WNT/β‐catenin pathway, which suppresses *CEACAM5* expression. Inhibition of WNT/β‐catenin signaling sensitized these cells to cibisatamab, indicating that CEA plasticity is regulated by drug selection and cytokine signaling.

Our study observed that EGFR‐TKI treatment selected for LUAD cells exhibiting EMT characteristics, accompanied by reduced *CEACAM5* expression. EMT, a process regulated by cytokines and epigenetic factors, plays a critical role in cancer metastasis and the development of acquired resistance to EGFR‐TKI [50, 51, 52, 53]. We further found that treatment with romidepsin, a histone deacetylase inhibitor, induced EMT and downregulated CEACAM5 expression, suggesting the involvement of HDAC1/2 in both EMT and CEACAM5 regulation. Additionally, TGF‐β treatment downregulated CEACAM5 expression and induced EMT in both *EGFR*‐mutated and wild‐type LUAD cells, highlighting the role of TGF‐β in regulating CEACAM5 plasticity. Interestingly, CEACAM5 overexpression has been reported to inhibit invasion in breast cancer cells and block TGF‐β‐mediated EMT [39, 54]. While EMT promotes cancer dissemination, it often reduces cancer cell proliferation, indicating a transition from a proliferative state to an invasive mode [52]. At metastatic sites, reverse EMT (rEMT) can enable cancer cells to regain proliferative capacity [55, 56]. Whether serum CEACAM5 fluctuations reflect EMT and rEMT in primary tumors and metastatic sites to enable cancer cell dissemination and proliferation under EGFR‐TKI treatment and latter drug resistance requires further investigation. Our findings raise the question of whether fluctuations in serum CEACAM5 reflect EMT and rEMT processes in both primary tumors and metastatic sites, contributing to cancer dissemination and drug resistance under EGFR‐TKI treatment. This highlights the importance of understanding CEA heterogeneity and plasticity in cancer cells for optimizing treatment, particularly in the context of targeted therapies and chemotherapy. Further research is needed to elucidate these dynamics, which could lead to more effective strategies for overcoming resistance in advanced‐stage lung cancers.

In conclusion, the distinct prognostic significance of baseline CEA levels and their heterogeneity trends at relapse, as observed between EGFR‐TKI therapy and chemotherapy, highlights the clinical importance of CEA in different therapeutic contexts. By utilizing baseline CEA and monitoring CEA dynamics, clinicians can improve patient outcomes through more personalized and effective treatment strategies, ultimately enabling more precise and individualized care.

## Author Contributions

**Yen‐Shou Kuo:** conceptualization, data curation, writing – original draft, writing – review and editing, funding acquisition, investigation. **Ming‐Yi Zheng:** investigation, data curation, writing – review and editing. **Yi‐Shing Shieh:** conceptualization, writing – review and editing, writing – original draft, supervision. **Tsai‐Wang Huang:** conceptualization, writing – original draft, writing – review and editing, supervision. **Yu‐Ting Chou:** conceptualization, writing – review and editing, writing – original draft, funding acquisition, supervision, data curation.

## Conflicts of Interest

The authors declare no conflicts of interest.

## Supporting information


**Figure S1:** Correlation between CEA expression and PFS in patients with advanced‐stage LUAD treated with different first‐line modalities.
**Figure S2:** Prognostic impact of first‐line EGFR‐TKI gefitinib use on survival outcomes. Kaplan–Meier curves comparing first‐line gefitinib versus other EGFR‐TKIs in patients with *EGFR*‐mutant lung cancer. Panel A: Overall survival (log‐rank *p* = 0.014); panel B: Recurrence‐free survival (log‐rank *p* = 0.992).
**Figure S3:** Effect of baseline CEA expression on the prognostic impact of first‐line gefitinib treatment. Kaplan–Meier analysis of overall survival in *EGFR*‐mutant patients treated with first‐line gefitinib and high baseline CEA levels (≥ 5 ng/mL), compared to those receiving other EGFR‐TKIs with either high or low CEA levels (log‐rank *p* = 0.192).
**Figure S4:** Distinct CEA heterogeneity patterns in LUAD patients under first‐line EGFR‐TKI therapy versus chemotherapy.
**Table S1:** Association between clinical characteristics and OS or PFS in all 284 patients with advanced‐stage LUAD.
**Table S2:** Association between clinical characteristics and OS or PS in 102 patients with advanced‐stage LUAD treated with first‐line chemotherapy.
**Table S3:** Cox regression model including an interaction term between CEA level and first‐line gefitinib.

## Data Availability

The data that support the findings of this study are available on request from the corresponding author. The data are not publicly available due to privacy or ethical restrictions.
