# Correction to “Prognostic and Monitoring Utility of Serum CEA in Lung Adenocarcinoma: Differential Roles in EGFR‐TKI and Chemotherapy Treatments”

**DOI:** 10.1002/cam4.71830

**Published:** 2026-04-09

Y.‐S. Kuo, M.‐Y. Zheng, Y.‐S. Shieh, T.‐W. Huang, and Y.‐T. Chou, “Prognostic and Monitoring Utility of Serum CEA in Lung Adenocarcinoma: Differential Roles in EGFR‐TKI and Chemotherapy Treatments,” *Cancer Medicine* 14, no. 17 (2025): e71170, https://doi.org/10.1002/cam4.71170.

In the Affiliation section, the affiliation for author Yen‐Shou Kuo was incomplete. The text “Division of Thoracic Surgery, Department of Surgery, Tri‐Service General Hospital, National Defense Medical University, Taipei, Taiwan” was incorrect.

This should have read: “Division of Thoracic Surgery, Department of Surgery, Tri‐Service General Hospital, National Defense Medical University, Taipei, Taiwan; Graduate Institute of Medical Sciences, College of Medicine, National Defense Medical University, Taipei, Taiwan.”

We apologize for this error.

