# Supplementary material for: Prognostic and Monitoring Utility of Serum CEA in Lung Adenocarcinoma: Differential Roles in EGFR‐TKI and Chemotherapy Treatments
Source: Cancer Med. 2025 Aug 26;14(17):e71170. doi: 10.1002/cam4.71170 (PMC12378701; doi:10.1002/cam4.71170)
Supplement: Supplementary file 1 — Figure S1: Correlation between CEA expression and PFS in patients with advanced‐stage LUAD treated with different first‐line modalities. Figure S2: Prognostic impact of first‐line EGFR‐TKI gefitinib use on survival outcomes. Kaplan–Meier curves comparing first‐line gefitinib versus other EGFR‐TKIs in patients with EGFR‐mutant lung cancer. Panel A: Overall survival (log‐rank p = 0.014); panel B: Recurrence‐free survival (log‐rank p = 0.992). Figure S3: Effect of baseline CEA expression on the prognostic impact of first‐line gefitinib treatment. Kaplan–Meier analysis of overall survival in EGFR‐mutant patients treated with first‐line gefitinib and high baseline CEA levels (≥ 5 ng/mL), compared to those receiving other EGFR‐TKIs with either high or low CEA levels (log‐rank p = 0.192). Figure S4: Distinct CEA heterogeneity patterns in LUAD patients under first‐line EGFR‐TKI therapy versus chemotherapy. Table S1: Association between clinical characteristics and OS or PFS in all 284 patients with advanced‐stage LUAD. Table S2: Association between clinical characteristics and OS or PS in 102 patients with advanced‐stage LUAD treated with first‐line chemotherapy. Table S3: Cox regression model including an interaction term between CEA level and first‐line gefitinib. [file CAM4-14-e71170-s001.docx]

**Supplementary Information**

**Prognostic and Monitoring Utility of Serum CEA in Lung Adenocarcinoma: Differential Roles in EGFR-TKI and Chemotherapy Treatments**

Yen-Shou Kuo^1^, Ming-Yi Zheng^2^, Yi-Shing Shieh^3*^, Tsai-Wang Huang^1*^, Yu-Ting Chou^2*^

^1^Division of Thoracic Surgery, Department of Surgery, Tri-Service General Hospital, National Defense Medical University, Taipei, Taiwan.

^2^Institute of Biotechnology, National Tsing Hua University, Taiwan.

^3^Department of Dentistry, Tri-Service General Hospital, Taipei, Taiwan, National Defense Medical University, Taipei, Taiwan.


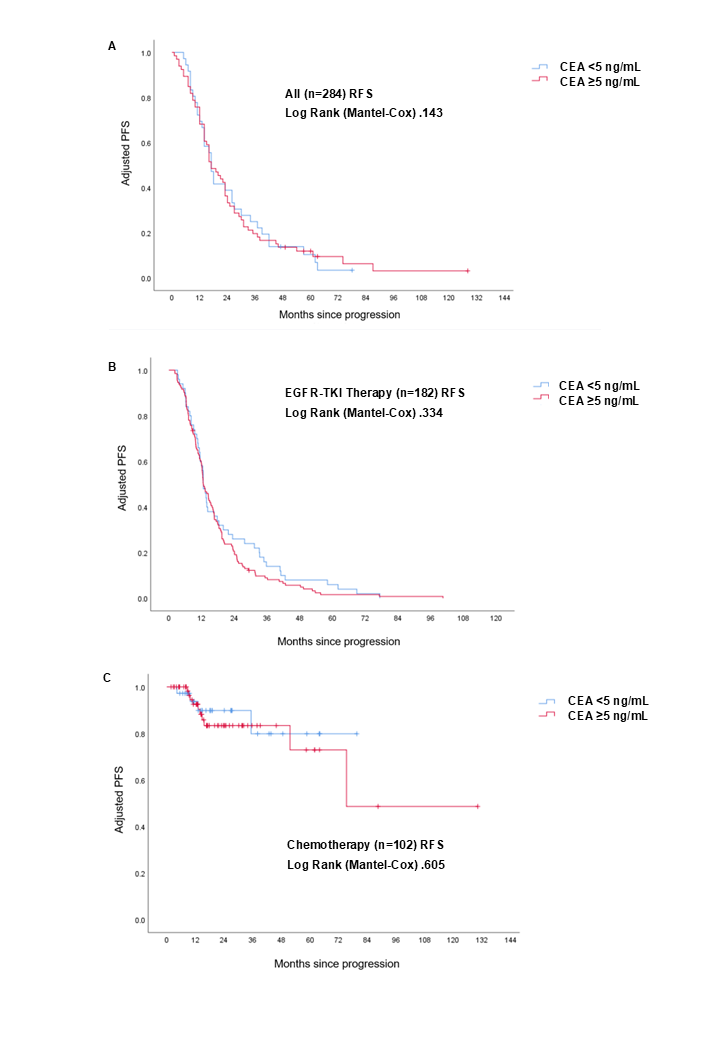


**Figure S1. Correlation between CEA expression and PFS in patients with advanced-stage LUAD treated with different first-line modalities**

This figure depicts adjusted PFS based on a multivariable Cox model, evaluating the relationship between CEA expression and different first-line treatments in patients with advanced-stage LUAD. Panel (A) includes all patients, while panel (B) focuses on those receiving EGFR-TKI therapy, and panel (C) on those receiving chemotherapy.

**Table S1. Association between clinical characteristics and OS or PFS in all 284 patients with advanced-stage LUAD**

|  | Overall survival | | | | Progression-free survival | | | |
| --- | --- | --- | --- | --- | --- | --- | --- | --- |
|  | Univariate analysis | | Multivariate analysis | | Univariate analysis | | Multivariate analysis | |
|  | HR(95%CI) | p | aHR(95%CI) | p | HR(95%CI) | p | aHR(95%CI) | p |
| Age ≧65 years | 1.108(0.866,1.420) | 0.415 | 1.190(0.927,1.527) | 0.173 | 1.460(1.096,1.945) | 0.010 | 1.271(0.947,1.704) | 0.110 |
| Male sex | 1.389(1.082,1.783) | 0.010 | 1.261(0.925,1.717) | 0.142 | 0.935(0.701,1.248) | 0.650 | 1.326(0.925,1.900) | 0.125 |
| Stage IV | 1.567(0.980,2.508) | 0.061 | 2.371(1.446,3.887) | 0.001 | 3.071(1.612,5.850) | 0.001 | 1.493(0.772,2.886) | 0.234 |
| Ever smoking | 1.331(1.026,1.728) | 0.031 | 1.077(0.778,1.490) | 0.656 | 0.827(0.602,1.135) | 0.239 | 1.136(0.766,1.684) | 0.525 |
| *EGFR* mutant | 0.588(0.455,0.760) | <0.001 | 0.511(0.387,0.674) | <0.001 | 9.759(5.658,16.831) | <0.001 | 9.731(5.523,17.145) | <0.001 |
| Baseline CEA |  |  |  |  |  |  |  |  |
| <5 ng/mL | Ref. |  | Ref. |  | Ref. |  | Ref. |  |
| ≥5 ng/mL | 1.192(0.907,1.566) | 0.207 | 1.199(0.911,1.578) | 0.194 | 1.265(0.923,1.734) | 0.145 | 1.160(0.842,1.597) | 0.364 |

HR, hazard ratio; aHR, adjusted hazard ratio; CI, confidence interval; EGFR, epidermal growth factor receptor; CEA, carcinoembryonic antigen level in serum at disease diagnosis.

**Table S2. Association between clinical characteristics and OS or PS in 102 patients with advanced-stage LUAD treated with first-line chemotherapy**

|  | Overall survival | | | | Progression-free survival | | | | |
| --- | --- | --- | --- | --- | --- | --- | --- | --- | --- |
|  | Univariate analysis | | Multivariate analysis | | Univariate analysis | | Multivariate analysis | | |
|  | HR(95%CI) | p | aHR(95%CI) | p | HR(95%CI) | p | aHR(95%CI) | p |  |
| Age ≧65 years | 1.315(0.876,1.976) | 0.186 | 1.362(0.906,2.048) | 0.137 | 6.023(1.663,21.808) | 0.006 | 5.803(1.597,21.080) | 0.008 |  |
| Male sex | 1.090(0.722,1.646) | 0.680 | 1.171(0.715,1.917) | 0.530 | 2.762(0.768,9.938) | 0.120 | 3.479(0.835,14.495) | 0.087 |  |
| Stage IV | 2.151(1.203,3.848) | 0.010 | 2.317(1.280,4.194) | 0.006 | 0.804(0.239,2.707) | 0.725 | 1.071(0.272,4.225) | 0.922 |  |
| Ever smoking | 1.015(0.677,1.523) | 0.942 | 1.049(0.643,1.709) | 0.849 | 1.130(0.395,3.233) | 0.819 | 0.557(0.150,2.063) | 0.381 |  |
| Baseline CEA |  |  |  |  |  |  |  |  |  |
| <5 ng/mL | Ref. |  | Ref. |  | Ref. |  | Ref. |  |  |
| ≥5 ng/mL | 0.977(0.655,1.520) | 0.991 | 0.980(0.641,1.497) | 0.924 | 1.357(0.425,4.340) | 0.606 | 1.271(0.383,4.215) | 0.695 |  |

HR, hazard ratio; aHR, adjusted hazard ratio; CI, confidence interval; EGFR, epidermal growth factor receptor; CEA, carcinoembryonic antigen level in serum at disease diagnosis.


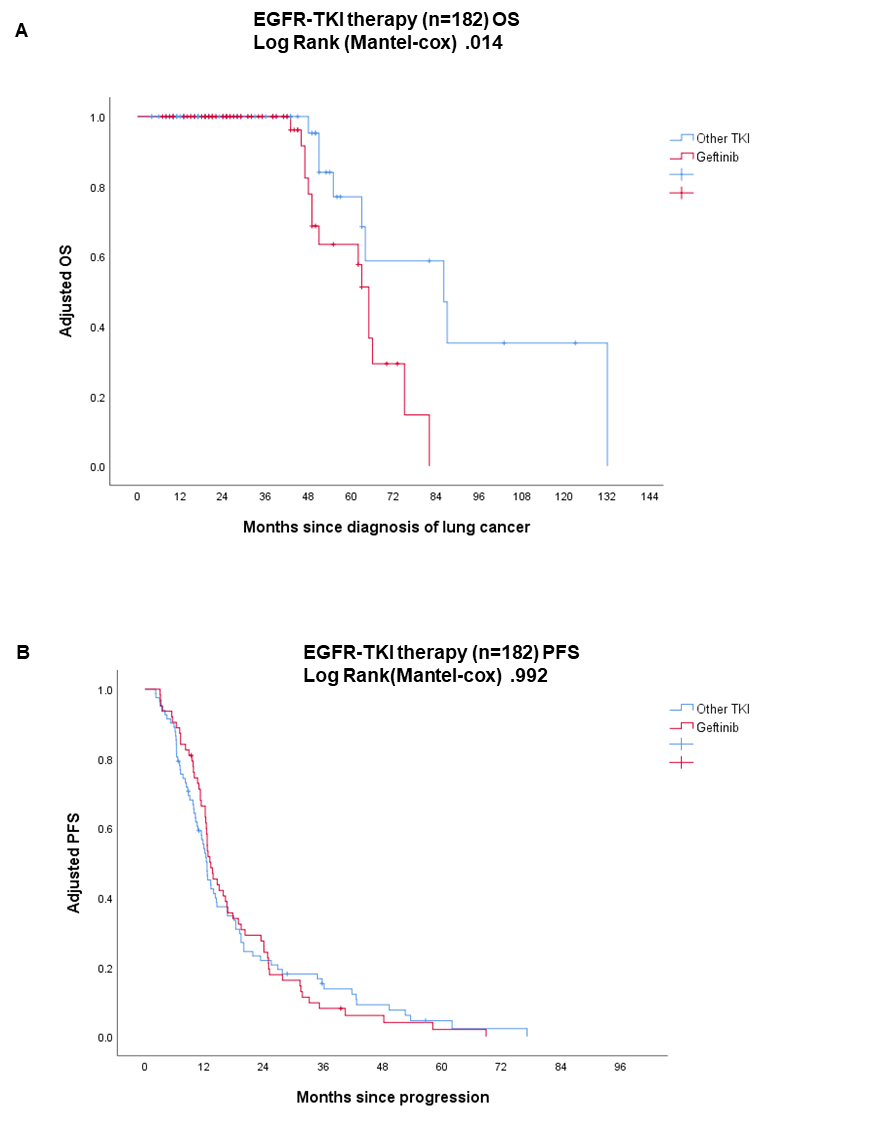


**Figure S2. Prognostic impact of first-line EGFR-TKI gefitinib use on survival outcomes.**Kaplan–Meier curves comparing first-line gefitinib versus other EGFR-TKIs in patients with *EGFR*-mutant lung cancer. Panel A: Overall survival (log-rank p = 0.014); panel B: Recurrence-free survival (log-rank p = 0.992).

**Table S3. Cox regression model including an interaction term between CEA level and first-line gefitinib**

| Variable | HR (Exp(B)) | p-value | 95% CI for HR |
| --- | --- | --- | --- |
| CEA (≥5 ng/mL)+Gef | 0.390 | 0.062 | 0.145 – 1.049 |

**
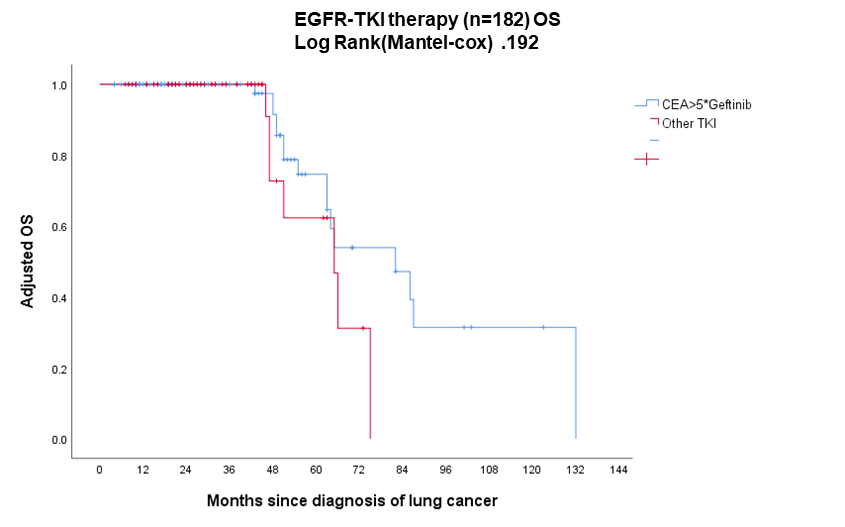
**

**Figure S3. Effect of baseline CEA expression on the prognostic impact of first-line gefitinib treatment.**Kaplan–Meier analysis of overall survival in *EGFR*-mutant patients treated with first-line gefitinib and high baseline CEA levels (≥5 ng/mL), compared to those receiving other EGFR-TKIs with either high or low CEA levels (log-rank p = 0.192).


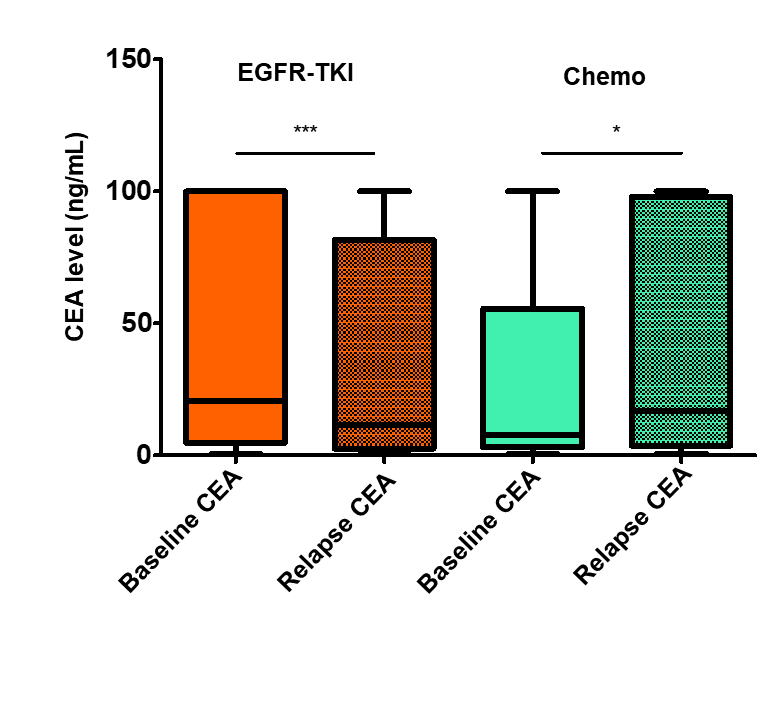


**Figure S4. Distinct CEA heterogeneity patterns in LUAD patients under first-line EGFR-TKI therapy versus chemotherapy**

This figure presents a box plot analysis of baseline and relapse CEA levels in patients treated with EGFR-TKI therapy (left, n=182) and chemotherapy (right, n=102). The Wilcoxon signed-rank test compared paired CEA levels within each treatment group.

**Supplementary Materials and Methods**

**Prognostic and Monitoring Utility of Serum CEA in Lung Adenocarcinoma: Differential Roles in EGFR-TKI and Chemotherapy Treatments**

Yen-Shou Kuo^1^, Ming-Yi Zheng^2^, Yi-Shing Shieh^3*^, Tsai-Wang Huang^1*^, Yu-Ting Chou^2*^

^1^Division of Thoracic Surgery, Department of Surgery, Tri-Service General Hospital, National Defense Medical University, Taipei, Taiwan.

^2^Institute of Biotechnology, National Tsing Hua University, Taiwan.

^3^Department of Dentistry, Tri-Service General Hospital, Taipei, Taiwan, National Defense Medical University, Taipei, Taiwan.

| **Primer** | **Sequence (5’-3’)** | **Probe** |
| --- | --- | --- |
| **CEACAM5_F** | **ACCACAGTCACGACGATCAC** | **UPL#9** |
| **CEACAM5_R** | **CTCCACGGGGTTGGAGTT** |  |
| **18S_F** | **TGGCTCATTAAATCAGTTATG** | **CGCTCGCTCCTCTCCTACTTG** |
| **18S_R** | **CGGCATGTATTAGCTCTA** |  |
| **CDH1_F** | **CAGGCTCAAGCTATCCTTGC** | **UPL#33** |
| **CDH1_R** | **AGTCATGCGTAGTGGTGCAT** |  |
| **VIM_F** | **TACAGGAAGCTGCTGGAAGG** | **UPL#15** |
| **VIM_R** | **CCAGAGGGAGTGAATCCAGA** |  |

**TaqMan qPCR primer and probe sequence list table**

| Cohort | Sources |
| --- | --- |
| GSE38310 | **GEO (1)** |
| GSE64322 | **GEO (2)** |

**Public domain database list table**

1. **https://www.ncbi.nlm.nih.gov/geo/query/acc.cgi?acc=GSE38310**
2. **https://www.ncbi.nlm.nih.gov/geo/query/acc.cgi?acc=GSE64322**
